# The subdued post-boost spike-directed secondary IgG antibody response in Ugandan recipients of the Pfizer-BioNTech BNT162b2 vaccine has implications for local vaccination policies

**DOI:** 10.3389/fimmu.2024.1325387

**Published:** 2024-02-16

**Authors:** Violet Ankunda, Joseph Ssebwana Katende, Gerald Kevin Oluka, Jackson Sembera, Claire Baine, Geoffrey Odoch, Peter Ejou, Laban Kato, The COVID-19 Immunoprofiling Team, Christine Hermilia Akoli, Pontiano Kaleebu, Jennifer Serwanga

**Affiliations:** ^1^Viral Pathogens Research Theme, Medical Research Council, Uganda Virus Research Institute and London School of Hygiene and Tropical Medicine, Uganda Research Unit, Entebbe, Uganda; ^2^Department of Immunology, Uganda Virus Research Institute, Entebbe, Uganda

**Keywords:** Pfizer BioNTech BNT162b2 COVID-19 vaccine, longitudinal antibody responses, Sub-Saharan African populations, seropositivity classification, booster dose, breakthrough infections, Ugandan population, IgG IgM and IgA antibodies

## Abstract

**Introduction:**

This study aimed to delineate longitudinal antibody responses to the Pfizer-BioNTech BNT162b2 COVID-19 vaccine within the Ugandan subset of the Sub-Saharan African (SSA) demographic, filling a significant gap in global datasets.

**Methods:**

We enrolled 48 participants and collected 320 specimens over 12 months after the primary vaccination dose. A validated enzyme-linked immunosorbent assay (ELISA) was used to quantify SARS-CoV-2-specific IgG, IgM, and IgA antibody concentrations (ng/ml) and optical densities (ODs). Statistical analyses included box plots, diverging bar graphs, and the Wilcoxon test with Bonferroni correction.

**Results:**

We noted a robust S-IgG response within 14 days of the primary vaccine dose, which was consistent with global data. There was no significant surge in S-IgG levels after the booster dose, contrasting trends in other global populations. The S-IgM response was transient and predominantly below established thresholds for this population, which reflects its typical early emergence and rapid decline. S-IgA levels rose after the initial dose then decreased after six months, aligning with the temporal patterns of mucosal immunity. Eleven breakthrough infections were noted, and all were asymptomatic, regardless of the participants’ initial S-IgG serostatus, which suggests a protective effect from vaccination.

**Discussion:**

The Pfizer-BioNTech BNT162b2 COVID-19 vaccine elicited strong S-IgG responses in the SSA demographic. The antibody dynamics distinctly differed from global data highlighting the significance of region-specific research and the necessity for customised vaccination strategies.

## Introduction

A global health crisis triggered by the Severe Acute Respiratory Syndrome Coronavirus 2 (SARS-CoV-2), responsible for the COVID-19 pandemic, necessitated rapid research and development of vaccines. Among these, the Pfizer-BioNTech COVID-19 vaccine (BNT162b2) showed significant efficacy in clinical trials, which were mainly undertaken in Western cohorts. A significant knowledge gap remains regarding the vaccine’s performance in Sub-Saharan Africa (SSA). The region’s distinctive genetic diversity, its widespread endemic diseases, and unique microenvironmental factors, highlight the necessity to assess the performance of these vaccines in this specific demographic. Collectively, these distinct factors have been shown to invariably impact responses to various viral vaccines, highlighting the critical need to understand the implications on COVID-19 vaccines within this specific demographic (1–6). In this study, we analysed the 12-month serological responses to the Pfizer-BioNTech BNT162b2 COVID-19 vaccine in a Ugandan cohort, revealing potential disparities and contributing crucial region-specific data from SSA. This study aimed at guiding the design of vaccination strategies and discerning trajectories of long-term immunity in the region. By examining the correlations between baseline antibody levels, breakthrough infections, and subsequent antibody responses, we aimed to provide insights to appropriately guide public health prevention strategies that aligning with the unique contexts of the region.

We provide a comprehensive one-year analysis of the SARS-CoV-2-specific IgG, IgM, and IgA antibody dynamics for a region typically underrepresented in global vaccine research. We show the elicited seroconversion patterns and how they are influenced by baseline serostatus, with broader implications for booster strategies and breakthrough infections. The findings contribute pivotal insights for refining vaccination strategies and public health policies throughout Africa by bridging a significant knowledge gap. The findings emphasize the importance of understanding diverse vaccine responses to facilitate the formulation of customized and pragmatic immunization strategies.

## Materials and methods

### Study population and study design

**Figure 1:**
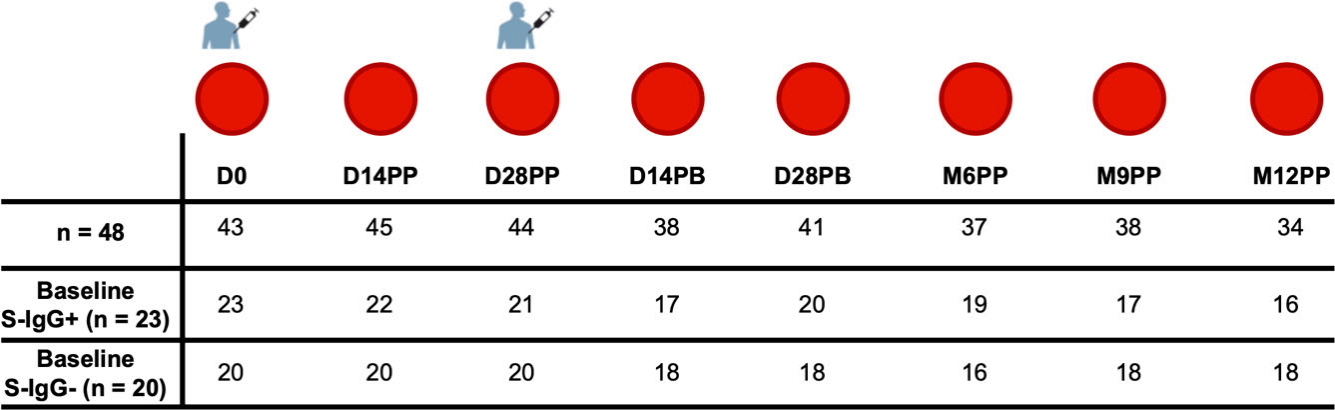
The Pfizer-BioNTech COVID-19 (BNT162b2) vaccine Administration and Sample Collection schedule. This figure illustrates vaccination and sample collection from 48 participants, detailing the number of samples obtained at each time point and the the number of subjects that were baseline S-IgG+ and baseline S-IgG-.

We obtained 320 specimens from 48 participants who were administered two doses of the Pfizer-BioNTech COVID-19 BNT162b2 vaccine, given initially on day 0 and followed by a second booster dose on day 30. Samples were collected over 12-months, from March 21, 2021, to January 6, 2023, to monitor the participants’ antibody responses after vaccination. In this study, 48 participants aged 19 to 49 years were analysed, comprising 22 females (45.8%) and 26 males (54.2%), with a median age of 30 and an interquartile range (IQR) of 25 to 35 years. Blood samples were collected immediately before administration of the first Pfizer-BioNTech BNT162b2 COVID-19 vaccine dose, and subsequently on days 14 (D14PP) and 28 D28PP) following the first dose. A booster dose was administered approximately 30 days after the primary vaccination, followed by sample collections at 14 (D14PB) and 28 days post-boost (D28PB), and then at six (M6PP), nine (M9PP), and twelve (M12PP) months after the primary dose. Of the 48 participants, we had baseline Spike protein-specific IgG (S-IgG) data for 43, enabling us to categorise the cohort based on their S-IgG seropositivity, as shown in **Figure 1** and summarised in **Table 1**.

**Table 1:** Demographic and Methodological Overview of the Study.

Parameter	Details
Total Subjects	48
Duration of Follow-Up	12 months (21st March 2021 to 6th January 2023)
Vaccine Administered	Pfizer-BioNTech BNT162b2 COVID-19 vaccine
Gender Distribution	Females: 22 (45.8%), Males: 26 (54.2%)
Age Range	19 - 49 years
Median Age	30 years (IQR: 25-35.3)
Baseline S-IgG Classification	43 subjects classified (20 S-IgG-, 23 S-IgG+)
S-IgG Cut-off OD for Baseline Classification	≥ 0.432 (Positive), < 0.432 (Negative)
Note	5 subjects had no day 0 (D0) sample

This table summarizes the demographics and baseline characteristics of the 48 participants enrolled in the study.

### Binding antibody ELISA to detect SARS-CoV-2-specific IgG, IgM, and IgA levels

We used a validated enzyme-linked immunosorbent assay (ELISA) protocol (7) to accurately measure SARS-CoV-2-directed antibodies, targeting both spike and nucleoprotein antigens. This allowed us to quantify IgG, IgM, and IgA concentrations in ng/ml based on detected optical densities at 450 nm. We coated the plates with 3 μg/ml antigen, equivalent to a total protein amount of 0.15 μg per well. This concentration was determined through a comprehensive assay validation, which involved testing a range of coating concentrations. The established cut-off values for antibody positivity within this population were: S-directed IgG (0.432), IgM (0.459), and IgA (0.226); and N-directed IgG (0.454), IgM (0.229), and IgA (0.225). Detailed steps regarding the ELISA protocol and cut-off criteria can be found in our previous publication (7, 8).

### Statistical methods

We used box plots to visually illustrate the key statistical metrics, including medians as horizontal lines within boxes), means represented as black dots inside the boxes, and quartiles defined by the top and bottom edge of boxes. Diverging bar graphs were used to show the percentage of seroconversion at various follow-up time points. We applied the Wilcoxon test for pairwise comparisons to determine significant differences in antibody responses across time points, with a Bonferroni correction to adjust for multiple testing. Given the occasional missing data/samples across time points, we opted for unpaired tests. A threshold of p > 0.05 was deemed non-significant (ns). Significance levels were as follows: * for p <= 0.05, ** for p < 0.01, *** for p < 0.001, and **** for p < 0.0001.

## Results

### Dynamic serological responses to primary vaccination: rapid and sustained seroconversion of S-IgG and S-IgA, with transient S-IgM seropositivity

**Figure 2:**
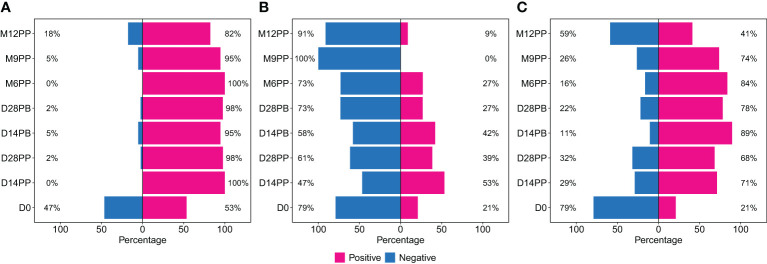
Rates of Spike Protein-Directed Seroconversion Following Pfizer-BioNTech COVID-19 (BNT162b2) Vaccination. This figure shows the percentage of participants who seroconverted at each time point. Using cutoffs of 0.432 for S-IgG, 0.459 for S-IgM, and 0.226 for S-IgA, the 320 samples were categorized as either positive or negative for IgG **(A)**, IgM **(B)** and IgA **(C)** antibodies. The bar graphs represent the proportion of participants with positive or negative status at each interval.

We utilised the pre-established population-specific seropositivity thresholds of 0.432 for S-IgG, 0.459 for S-IgM, and 0.226 for S-IgA, which were previously described (7). At the start of the study (baseline), participants were categorised into two groups as either serologically positive or negative for S-IgG antibodies based on these predefined cutoff values. We present data categorised by baseline S-IgG seropositivity, with participant counts and corresponding seropositivity percentages, reflecting the presence or absence of antibodies at specific time points. All 320 samples were included in the classification analysis, and the distribution of these samples based on S-IgG seropositivity at each time point is visually represented in **Figure 2**.

We began the study on day 0, with 47% of the participants lacking S-IgG antibodies. By day 14 after the initial vaccination, all participants showed S-IgG positivity; these levels persisted above 80% throughout the subsequent study duration, as depicted in **Figure 2A**. The S-IgM antibodies initially appeared in 21% of participants on day 0, reaching a peak of 53% on day 14 post-primary vaccination. However, most participants saw their antibody levels decline, reaching seropositivity rates of 27% six months after the primary dose and eventually dropping to zero nine months the primary vaccination, as shown in **Figure 2B**. The proportion of individuals with detectable S-IgA antibodies exhibited a notable increase post-vaccination, rising from 21% at baseline to 71% two weeks after the initial dose. This trend continued, reaching a peak of 89% two weeks following the booster dose. Subsequent levels of IgA seropositivity remained consistently high, above 70% for an extended period before declining to 41% at the 12-month mark (**Figure 2C**).

Median nucleoprotein-directed IgG (N-IgG) antibodies remained predominantly suboptimal throughout the study, showing a non-significant increase only at the 12-month mark, regardless of the baseline S-IgG serostatus (**Supplementary Figures 1A, B**). Initially, positive N-IgG responses were detected in only 26% of the participants. This proportion remained relatively stable, hovering around 20% until 28 days post-booster dose. Subsequently, there was a gradual increase to 38% at six months, escalating further to 62% by 12 months, as represented in **Supplementary Figure 1C**. Alongside this, N-IgM antibody levels were consistently low across all participants, irrespective of baseline S-IgG seropositivity status (**Supplementary Figures 1D, E**). The proportion of S-IgM seropositive participants gradually decreased from 51% at baseline to 24% on D28PB, surged to 57% at six months, suggesting possible breakthrough infections, and then sharply fell to 6% by 12 months (**Supplementary Figure 1F**).

### Post-vaccination trends revealed strong S-IgG response, limited impact of booster dose, suboptimal S-IgM Levels, and transient S-IgA elevation

**Figure 3:**
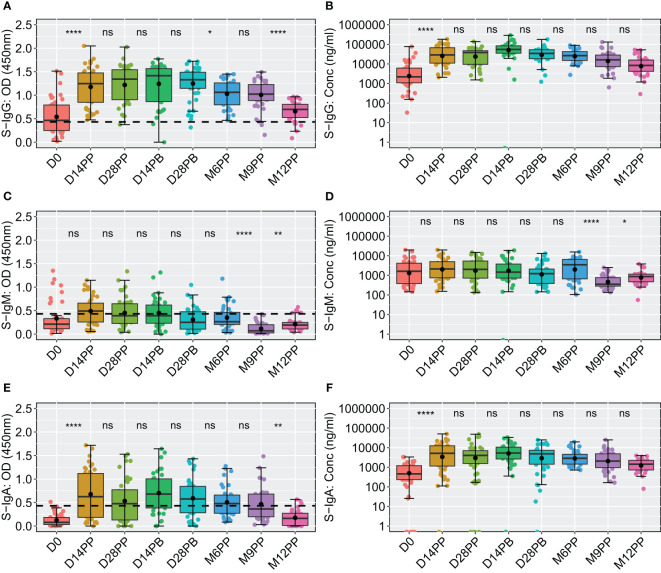
Comparative Analysis of Spike-Directed Antibody Responses: Boxplot Representation Across Different Time Points. This figure shows the distribution of antibody responses over time using boxplots, highlighting the median (lines), mean (black circles), and quartiles for IgG **(A, B)**, IgM **(C, D)** and IgA **(E, F)**. Significant differences between adjacent time points were assessed using an unpaired Wilcoxon test with a Bonferroni correction for multiple testing. Notation ns denotes p-values > 0.05 and was considered insignificant. *p ≤ 0.05, **p < 0.01, and ****p < 0.0001, indicating increasing levels of statistical significance.

We next examined the dynamics of Spike-directed antibodies, observing a significant increase in S-IgG levels from baseline to day 14 post-prime, sustained until 28 days post boost, after which a decline was evident, as determined by box plots and the unpaired Wilcoxon test, summarised in **Figure 3**. The baseline median OD values on day 0 were 0.464 with an interquartile range (IQR) of 0.250 to 0.793, corresponding to concentrations of 40.986 binding antibody units (BAU)/ml (IQR 20.314 to 113.864), as shown in **Figures 3A, B**, **Table 2**. Antibody levels surged to median OD levels of 1.247 and concentrations of 534.576 BAU/ml by day 14 post-prime (IQR: 0.850–1.474 and 204.062–1257.831, respectively), and remained elevated at day 28 post-boost, with OD values of 1.330 (IQR 1.147, 1.489) and concentrations of 642.461 BAU/ml (IQR 341.770, 993.056). S-IgG OD levels significantly declined between six to twelve months post-prime, as detailed in **Figure 3A** and **Table 2**. Following the initial surge, S-IgG antibody concentrations remained statistically stable throughout the follow-up period, illustrated in **Figure 3B**. In contrast, S-IgM antibody responses consistently exhibited suboptimal trends over this duration, as depicted in **Figures 3C, D**. Significant increases in S-IgA antibody levels, as indicated by elevated ODs and concentrations, were observed 14 days after the initial dose, persisting for nine months post-prime, followed by a notable decline in OD levels beyond nine months (**Figures 3E, F**). Nucleoprotein-directed antibody levels (N-IgG) were predominantly suboptimal, displaying a non-significant increase at month 12, while median N-IgM levels consistently remained low throughout the study period, **Supplementary Figure 2**.

**Table 2:** displaying the median S-directed antibody responses, including interquartile ranges (IQR), at each specified time point.

Time	Antibody	OD (450nm)	Conc (ng/ml)	Conc (BAU/ml)
D0	S-IgG	0.464 (0.250, 0.793)	2184.1 (1080.35, 6075.25)	40.986 (20.314, 113.864)
	S-IgM	0.214 (0.116, 0.331)	1623.9 (381.45, 4173.60)	60.382 (14.539, 154.458)
	S-IgA	0.078 (0.031, 0.183)	463.5 (235.05, 1206.25)	88.423 (44.824, 230.178)
D14PP	S-IgG	1.247 (0.850, 1.474)	28538.4 (10891.23, 67155.23)	534.576 (204.062, 1257.831)
	S-IgM	0.493 (0.259, 0.661)	2122 (751.3, 5005.1)	78.761 (28.186,185.138)
	S-IgA	0.626 (0.184, 1.119)	5162.6 (1153.45, 12481.15)	985.251 (220.101, 2382.004)
D28PP	S-IgG	1.343 (0.905, 1.542)	38004.55 (9502.225, 47145.775)	711.868 (178.048, 883.074)
	S-IgM	0.391 (0.227, 0.647)	2033.95 (721.275, 5369.500)	75.512 (27.078, 198.584)
	S-IgA	0.476 (0.129, 0.772)	4045.15 (1571.981, 7056.050)	771.985 (299.978, 1346.618)
D14PB	S-IgG	1.414 (0.864, 1.565)	54715.07 (34954.07, 83835.65)	997.875 (647.834, 1554.335)
	S-IgM	0.392 (0.232, 0.620)	1468.8 (733.9, 3802.9)	51.110 (25.645, 139.399)
	S-IgA	0.680 (0.385, 1.003)	5402.85 (2669.85, 10605.22)	1031.103 (509.508, 2023.982)
D28PB	S-IgG	1.330 (1.147, 1.489)	34298.7 (18243.85, 53018.05)	642.461 (341.770, 993.056)
	S-IgM	0.251 (0.107, 0.477)	1226.2 (387.6, 2107.5)	45.708 (14.766, 78.226)
	S-IgA	0.575 (0.282, 0.844)	4907.825 (1450.475, 7367.850)	936.627 (276.789, 1406.126)
M6PP	S-IgG	1.062 (0.797, 1.261)	26442.3 (15090.95, 42729.65)	495.3183 (282.719, 800.364)
	S-IgM	0.265 (0.206, 0.465)	3479.2 (662.1, 6469.9)	128.8372 (24.895, 239.185)
	S-IgA	0.478 (0.273, 0.659)	2880.3 (1459.8, 4616.7)	549.6722 (278.568, 881.066)
M9PP	S-IgG	1.024 (0.887, 1.227)	16081.6 (7290.45, 28502.82)	301.273 (136.6233, 533.910)
	S-IgM	0.071 (0.026, 0.201)	351.775 (280.500, 769.075)	13.445 (10.815, 28.842)
	S-IgA	0.363 (0.197 0.680)	2084.2 (982.075, 4875.062)	397.736 (187.394, 930.375)
M12PP	S-IgG	0.698 (0.523, 0.808)	8353.6 (4156.9, 15660.9)	156.535 (77.935, 293.393)
	S-IgM	0.195 (0.112, 0.249)	868.55 (473.275, 1158.550)	32.512 (17.928, 43.212)
	S-IgA	0.164 (0.014, 0.271)	1445.4 (727.775, 2257.650)	275.820 (138.861, 430.839)

This table displays the median S-directed antibody responses at various time points post-vaccination, using three different measures: Optical Density (OD) at 450nm, Concentration in ng/ml, and Concentration in Binding Antibody Units (BAU)/ml. The data is presented for three antibody types: S-IgG, S-IgM, and S-IgA, across multiple time points: Day 0 (D0), 14 days post-primary vaccination (D14PP), 28 days post-primary vaccination (D28PP), 14 days post-booster (D14PB), 28 days post-booster (D28PB), six months post-primary vaccination (M6PP), nine months post-primary vaccination (M9PP), and 12 months post-primary vaccination (M12PP). Interquartile ranges (IQR) are provided alongside each median value to indicate data variability.

**Figure 4:**
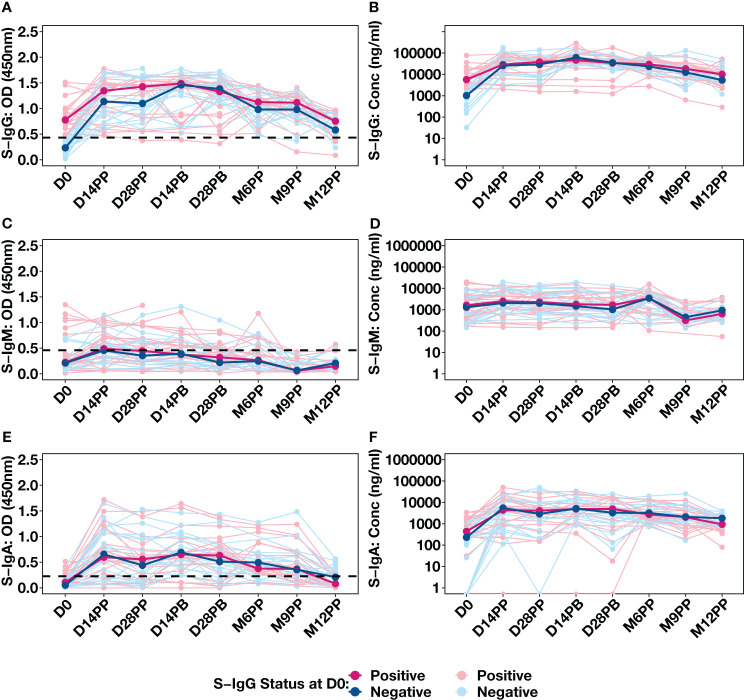
Individual Antibody Response Profiles Categorized by Baseline Spike Protein IgG Seropositivity. This figure shows the antibody responses against the Spike protein, including IgG **(A, B)**, IgM **(C, D)**, and IgA **(E, F)**. Participants were colour-coded based on S-IgG seropositivity at baseline: positive in pink (S-IgG levels ≥ S-IgG cutoff) and negative in blue. Thick lines indicate group median values, while thin lines depict individual profiles.

The availability of baseline S-IgG serostatus data for 43 individuals, allowed us to stratify our analysis based on their initial S-IgG seropositivity. Individuals were categorised as either S-IgG+ (in red) if their levels met or exceeded the S-IgG cutoff or as S-IgG- (in blue) if their levels fell below the threshold, as shown in **Figure 4**. There was a significant increase in S-IgG antibody responses 14 days after the initial dose, observed in both S-IgG positive and negative groups. While baseline antibody levels at D0 significantly differed between the two groups, with S-IgG+ participants displaying higher responses, the distributions of S-IgG antibody OD levels and concentrations after vaccination remained statistically indistinguishable (**Figures 4A, B**). After 14 days of priming, the S-IgG antibody concentrations remained comparable between both groups with no discernible enhancement in immune responses after the booster dose administration. Throughout the study, we consistently observed low S-IgM antibody responses below the established cutoff, with no distinguishable differences in S-IgM antibody OD levels (**Figures 4C**) and concentrations (**Figure 4D**) between participants who initially tested positive for S-IgG and those who did not. S-IgA antibody levels showed an initial increase within the first 14 days after priming, maintaining this elevation until 28 days post-boost, followed by a gradual decline. Notably, the responses did not significantly differ between participants with baseline S-IgG antibodies (S-IgG+) and those without (S-IgG-) at any assessed time points, as depicted in **Figures 4E, F**. The data show a robust S-IgG response post-vaccination, suboptimal S-IgM, and gradually declining moderate S-IgA responses after six months, highlighting key immunological dynamics.

### Longitudinal analysis of post-vaccination breakthrough infections reveals no significant differences based on baseline S-IgG serostatus

**Figure 5:**
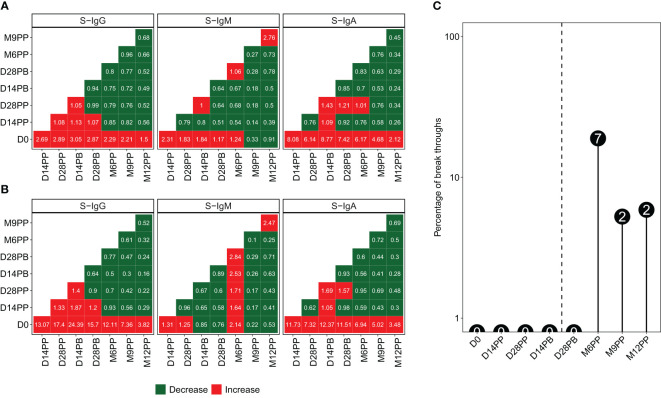
Variations in anti-Spike Antibody Levels and Incidence of Breakthrough Infections Following Pfizer-BioNTech COVID-19 (BNT162b2) Vaccination. Median antibody level changes over time, presented as optical density (OD) levels **(A)** and concentrations **(B)**, using red for increases and green for decreases, alongside the percentage and number of breakthrough cases at each time point **(C)**.

We generated heatmaps illustrating the median fold-changes in Spike-directed antibody responses between consecutive timepoints, based on pairwise analyses of antibody OD levels (**Figure 5A**) and concentrations (**Figure 5B**). Increased responses are presented in red, while reduced responses are depicted in green. Significant fold increases in S-IgG and S-IgA antibody responses were observed 14 days after the baseline (D0), with minimal changes observed between subsequent time points. There were minimal fluctuations in S-IgM levels across pairwise time points. In various studies, methods to distinguish reinfection from initial infection have shown consistency. A macaque study identified a 7.6-fold increase in N-IgG antibody levels as indicative of reinfection (9), paralleled by a human study in West Africa suggesting a 7-fold rise (10). Similar patterns of titre increase were also observed in high-income settings (11, 12). A recent and more controlled hospital-based study in Spain defined *reinfections* as a rise of at least 5.9% in titres of anti-nucleoprotein antibodies and/or a minimum of 5.1% increase in anti-RBD titres, provided there was no booster vaccination in the preceding three months (13). Here, we used an 11-fold increase in N-IgG levels, which was previously established as the threshold for presuming an infection in this population. Consequently, an 11-fold increase in N-IgG antibody levels after a completed vaccination regimen served as an indicative marker for potential breakthrough infection. Individuals who exhibited this surge in antibody levels within 14 days of their final vaccine dose were categorized as breakthrough cases. Eleven breakthrough infections were observed at varying time intervals following completion of the full vaccination course: seven cases emerged at six months post-vaccination, while two occurred at nine months and two at one-year post-primary vaccination, as depicted in **Figure 5C**. Excluding breakthrough infections, S-IgG antibody concentrations significantly increased 14 days post-prime, remaining stable after that. S-IgM remained suboptimal, while S-IgA notably rose until day 14, stabilising slightly above the threshold, as shown in **Supplementary Figure 3**.

### The early rise in S-IgG antibody responses in initially seronegative participants aligned with seropositive counterparts in the subsequent study duration

**Figure 6:**
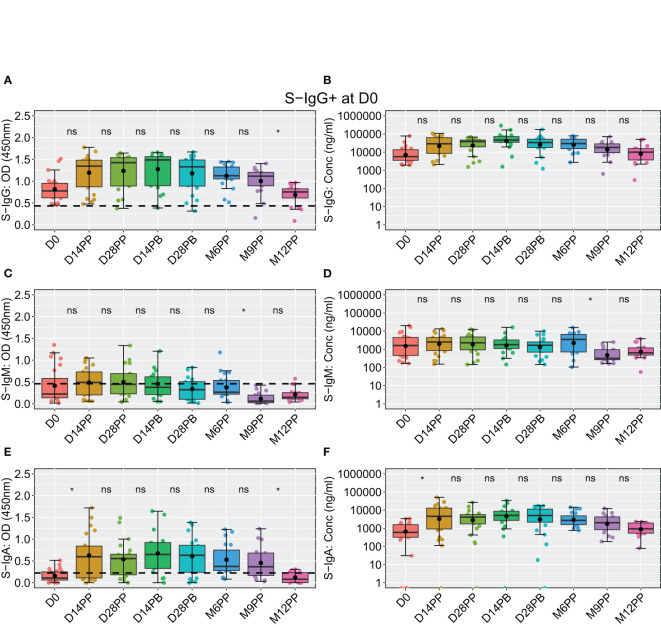
Longitudinal Analysis of BNT162b2-Induced Spike-Specific Antibody Responses in Baseline S-IgG Seropositive Individuals Over 12 months. Boxplots visually summarizing for 23 individuals that were seropositive for spike-directed IgG antibodies at baseline displaying temporal dynamics of spike-directed IgG **(A, B)**, IgM **(C, D)**, and IgA **(E, F)** antibody responses over the 12 months of follow up. in baseline S-IgG+ participants, with median lines, mean as black circles, and quartiles at box edges. Differences between time points were assessed using an unpaired Wilcoxon test with Bonferroni correction, denoted as ns (p > 0.05) and *(p ≤ 0.05).

At the outset of the study (D0), among 48 participants, 23 exhibited S-IgG seropositivity, 20 were seronegative, and five lacked baseline specimens. Initially, individuals with baseline S-IgG seropositivity displayed high antibody concentrations, which remained elevated and generally stable over time (**Figure 6A**), with a notable significant decline in OD levels observed at 12 months post-prime compared to 9 months, depicted in **Figure 6B**. These participants exhibited suboptimal levels of S-IgM (**Figures 6C, D**), with a moderate increase in S-IgA from D0 to D14PP, which persisted until M9PP before a declining significantly, as illustrated in **Figures 6E, F**.

**Figure 7:**
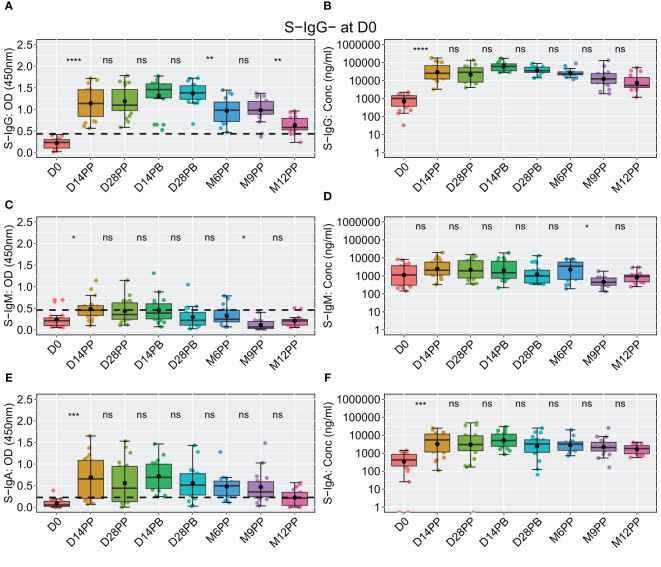
Comprehensive Analysis of the Pfizer-BioNTech BNT162b2 Vaccine-induced Spike-Directed Antibody Responses in S-IgG- Participants Over Time. Illustrates the dynamics of the Pfizer-BioNTech BNT162b2 vaccine-induced Spike-directed antibody responses (IgG, IgM, IgA) in 20 participants initially lacking anti-spike IgG (S-IgG) at baseline (Day 0). Panels **(A–F)** show the distribution of antibody optical density (OD) levels and concentrations in ng/ml across time points for IgG **(A, B)**, IgM **(C, D)**, and IgA **(E, F)**. Statistical analysis involved the Wilcoxon test for unpaired pairwise comparisons, with Bonferroni correction for multiple testing, to address missing data points. Boxplots display median (line), mean (black circle), and quartile values (box edges). Significance levels are indicated as: ns (p > 0.05), *(p ≤ 0.05), **(p < 0.01), ***(p < 0.001), ****(p < 0.0001).

Among the 20 participants lacking S-IgG antibodies at baseline, a significant rise in S-IgG levels was observed two weeks post-vaccination, with concentrations remaining statistically stable through the study period, including after boosting; however, at the six-month mark, a noticeable decline in optical density (OD) levels became evident, as depicted in **Figures 7A, B**. These participants consistently demonstrated suboptimal levels of S-IgM antibodies throughout the observation period (**Figures 7C, D**). At the same time, S-IgA responses exhibited an initial increase from D0 to D14 after the initial vaccination, followed by a gradual decline from two weeks after the booster shot and throughout subsequent visits, as indicated by optical densities and concentration measurements (**Figures 7E, F**).

## Discussion

In the global response to the SARS-CoV-2 pandemic, the Pfizer-BioNTech COVID-19 Vaccine (BNT162b2) was pivotal, showing notable efficacy in global populations (14). Despite its effectiveness, data on the Pfizer-BioNTech COVID-19 (BNT162b2) vaccine’s performance in the genetically diverse and distinct African demographic (15–17), with its prevalent activated microenvironments (1, 4, 18), known to impact vaccine-induced immunity (6, 19), remains notably scarce. This study was initiated in March 2021 alongside the first rollout of COVID-19 vaccines in Uganda. We navigated the pragmatic challenges of a national mass vaccination campaign during a lockdown, which prioritised broad coverage over the assessment of prior infections. Recognising established data indicating the more extended durability of spike-directed IgG antibodies compared to N-IgG antibodies in this population (20) and elsewhere (13), we used baseline levels of both as proxies for prior exposure, acknowledging earlier studies revealing low pre-epidemic cross-reactivity in this population (21). This enabled us to examine the enhancement of spike-directed antibody responses in the Ugandan population following vaccination, offering a realistic depiction of the vaccine’s performance in this demographic under real-life conditions. The rapid spike-directed IgG response within two weeks of the initial vaccine dose concurred with global trends and highlighted the vaccine’s effectiveness across varied demographics (22). We noted consistent levels of S-IgG antibodies after boosting, aligning with some populations (23), and with other vaccine types administered in this population (24, 25), but contrasts with others where levels distinctly declined 4-6 months post-vaccination (26–28). The antibody persistence in our Ugandan cohort suggests a potential benefit in modifying the boosting interval, as supported by mouse models (29), and the need to optimise vaccination strategies in this population.

The observed disparities may stem from factors such as genetic variations, unique HLA patterns in the African population affecting antigen processing (15), previous SARS-CoV-2 exposure potentially conferring immunity (30), and the consequences of premature boosting. The transient, subdued S-IgM response observed aligns with its typical early appearance and subsequent decline in favor of memory responses, as evidenced in natural infections within this population (20) and others (31, 32). S-IgA, essential in mucosal defence against SARS-CoV-2 (33), initially rose but declined by six months post-prime, providing insights into the temporal dynamics of mucosal immunity (23, 34), which is a crucial role against SARS-CoV-2 (35, 36). Breakthrough infections, uniformly distributed across groups irrespective of initial S-IgG levels, were asymptomatic, suggesting the effectiveness of the elicited immunity.

This study offers valuable insights but is limited by its small sample size (n=48), which may not fully represent the diverse SSA demographic (37–39). In line with this, our study’s limited sample size precluded an extensive stratification by gender. However, based on previous research into natural COVID-19 infections in this study setting, there was no substantial evidence to suggest gender-based differences in immunogenicity (20). Secondly, only binding antibody responses were examined, without their functional capacity (40) or T-cell-mediated immunity (41, 42), both of which are crucial for sustained viral defence. Also, there remains a need to fully reveal the long-term immune memory or responsiveness to evolving SARS-CoV-2 variants. Investigating the T-cell and genetic influences on the induced immunity will be crucial for optimising vaccination strategies. Consideration of the interplay between prior infections and dominant SARS-CoV-2 strains will inform optimisation of vaccine protocols for the region.

In conclusion, this study highlighted the antibody responses to the Pfizer-BioNTech COVID-19 vaccine in SSA, a demographic previously underrepresented in global datasets. We observed a strong S-IgG response, with notable differences in S-IgM and S-IgA dynamics and responses relative to baseline S-IgG serostatus. A post-boost surge was absent, emphasizing the need to re-evaluate the boosting strategies. Region-centric studies are vital to ensure that vaccination strategies resonate with diverse serological, environmental, and genetic contexts.

## Data availability statement

The raw data supporting the conclusions of this article will be made available by the authors, without undue reservation.

## Ethics statement

The studies involving humans were approved by Uganda Virus Research Institute’s Research and Ethics Committee (GC/127/833) and the Uganda National Council for Science and Technology (HS637ES). The studies were conducted in accordance with the local legislation and institutional requirements. The participants provided their written informed consent to participate in this study.

## Author contributions

VA: Formal analysis, Validation, Writing – original draft, Writing – review & editing, Data curation, Software. JK: Data curation, Writing – review & editing, Investigation, Methodology. GKO: Data curation, Investigation, Methodology, Writing – review & editing. JaS: Investigation, Methodology, Writing – review & editing. CB: Writing – review & editing, Data curation, Investigation, Methodology. GO: Writing – review & editing, Investigation, Methodology. PE: Writing – review & editing, Investigation, Methodology. LK: Data curation, Investigation, Methodology, Writing – review & editing. PK: Conceptualization, Funding acquisition, Supervision, Writing – original draft, Writing – review & editing. JeS: Conceptualization, Data curation, Formal analysis, Funding acquisition, Investigation, Project administration, Resources, Supervision, Validation, Visualization, Writing – original draft, Writing – review & editing.

## The COVID-19 immunoprofiling team

Christine Hermilia Akoli^1^, Angela Namuyanja^1^, Solomon Opio^1^, Arthur Watelo Kalyebi^1^, Ivan Ssali^1^, Ben Gombe^1^, Susan Mugaba^1^ and Hellen Nantambi^2^.

## Supplementary material

Supplementary Figure 1 Profile Plots Depicting Individual N-directed Responses of Antibodies and Seroconversion Across Different Time Points. **Supplementary Figure 1** displays the profiles of nucleoprotein-directed IgG and IgM binding antibody responses among study participants, stratified by baseline spike-directed IgG seropositivity. Baseline S-IgG-positive individuals (S-IgG+) are represented in red, while S-IgG-negative individuals (S-IgG-) are in blue. Line graphs show individual participant profiles (light-coloured lines) along with the group median (thick, dark-coloured lines). Divergent bar graphs show the percentage of seroconversion at each follow-up time point. **(A–C)** illustrate IgG responses, while **(D–F)** represent IgM responses. Median antibody levels for S-IgG+ and S-IgG- baseline participants are compared in the line graphs, while N-seroconversion frequencies are highlighted in the diverging bar graphs.

Supplementary Figure 2 Box plots illustrating the Longitudinal Assessment of Nucleoprotein Antibody Responses After Vaccination with no Breakthrough Participants. **Supplementary Figure 2**. illustrates the temporal profile of N-directed antibody responses across 12 months in the cohort. **(A, B)** display Nucleoprotein IgG optical density (OD) and concentrations, while IgM levels are depicted in **(C, D)**. Data distributions are summarised using boxplots, with medians represented by lines, means indicated by black circles, and quartiles marked by box edges. Significance between consecutive time points was determined using an unpaired Wilcoxon test with Bonferroni correction, denoting non-significant differences as “ns” (p > 0.05), ** (p < 0.01).

Supplementary Figure 3 Box Plots Demonstrating Longitudinal Spike-Directed Antibody Responses Post-Vaccination, Excluding Breakthrough Cases. **Supplementary Figure 3** depicts the temporal profiles of antibody responses in Pfizer-BioNTech COVID-19 (BNT162b2) vaccine recipients without breakthrough infections over 12 months. **(A, B)** illustrate spike-directed IgG optical density (OD) levels and concentrations, respectively, while **(C, D)** show IgM antibodies, and **(E, F)** represent IgA. Boxplots are used to summarise the data distributions, with medians indicated by lines, means represented by black circles, and quartiles by box edges. Statistical significance between consecutive time points was assessed using an unpaired Wilcoxon test with Bonferroni correction, denoted as follows: (ns: p > 0.05; *: p ≤ 0.05; **: p < 0.01; ***: p < 0.001; ****: p < 0.0001).
