# Supplementary material for: The subdued post-boost spike-directed secondary IgG antibody response in Ugandan recipients of the Pfizer-BioNTech BNT162b2 vaccine has implications for local vaccination policies
Source: Front Immunol. 2024 Feb 16;15:1325387. doi: 10.3389/fimmu.2024.1325387 (PMC10926532; doi:10.3389/fimmu.2024.1325387)
Supplement: Supplementary Figure 1 — Profile Plots Depicting Individual N-directed Responses of Antibodies and Seroconversion Across Different Time Points. Supplementary Figure 1 displays the profiles of nucleoprotein-directed IgG and IgM binding antibody responses among study participants, stratified by baseline spike-directed IgG seropositivity. Baseline S-IgG-positive individuals (S-IgG+) are represented in red, while S-IgG-negative individuals (S-IgG-) are in blue. Line graphs show individual participant profiles (light-coloured lines) along with the group median (thick, dark-coloured lines). Divergent bar graphs show the percentage of seroconversion at each follow-up time point. (A–C) illustrate IgG responses, while (D–F) represent IgM responses. Median antibody levels for S-IgG+ and S-IgG- baseline participants are compared in the line graphs, while N-seroconversion frequencies are highlighted in the diverging bar graphs. [file Presentation_1.pdf]

SUPPLEMENTARY FIGURES

Supplementary Figure 1: Profile Plots Depicting Individual N-directed Responses of Antibodies and Seroconversion across Different Time Points

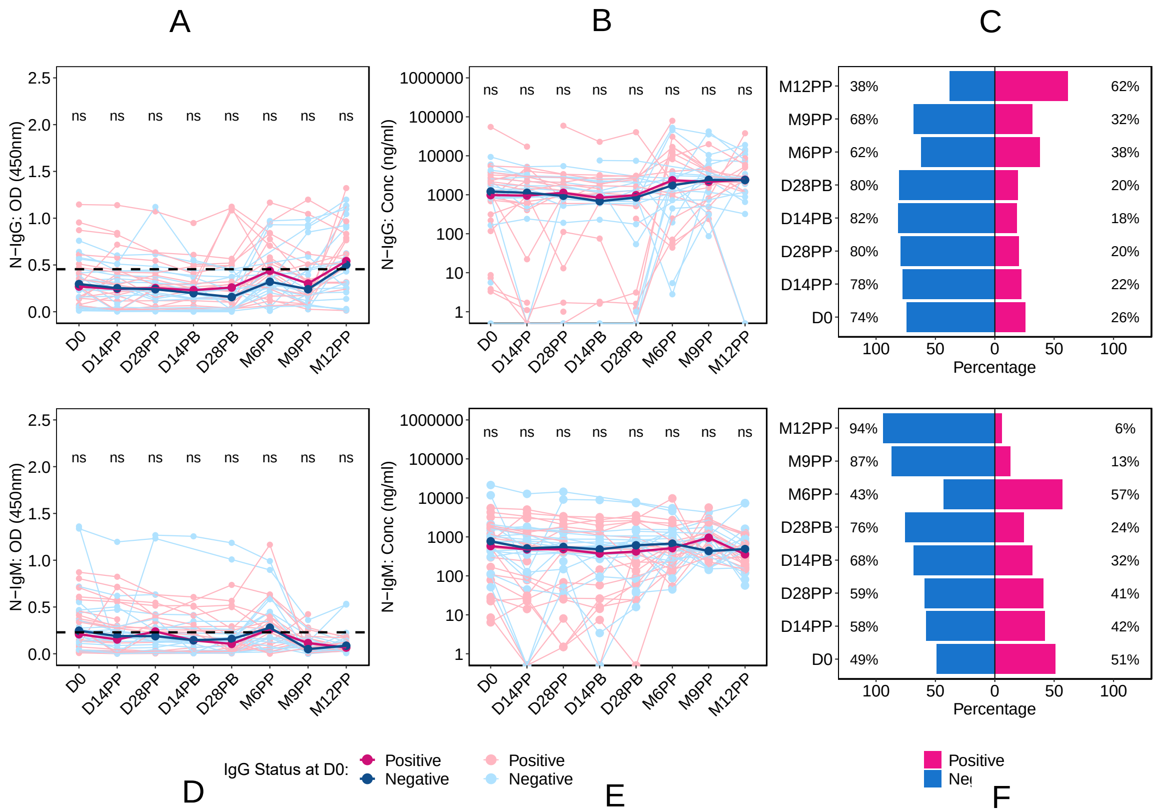

Supplementary Figure 1 displays the profiles of nucleoprotein-directed IgG and IgM binding antibody responses among study participants, stratified by baseline spike-directed IgG seropositivity. Baseline S-IgG-positive individuals (S-IgG+) are represented in red, while S-IgG-negative individuals (S-IgG-) are in blue. Line graphs show individual participant profiles (light-coloured lines) along with the group median (thick, dark-coloured lines). Divergent bar graphs show the percentage of seroconversion at each follow-up time point. Panels 1A, 1B and 1C illustrate IgG responses, while panels 1D, 1E, and 1F represent IgM responses. Median antibody levels for S-IgG+ and S-IgG- baseline participants are compared in the line graphs, while N-seroconversion frequencies are highlighted in the diverging bar graphs.

Supplementary Figure 2: Box plots illustrating the Longitudinal Assessment of Nucleoprotein Antibody Responses after Vaccination with no Breakthrough Participants.

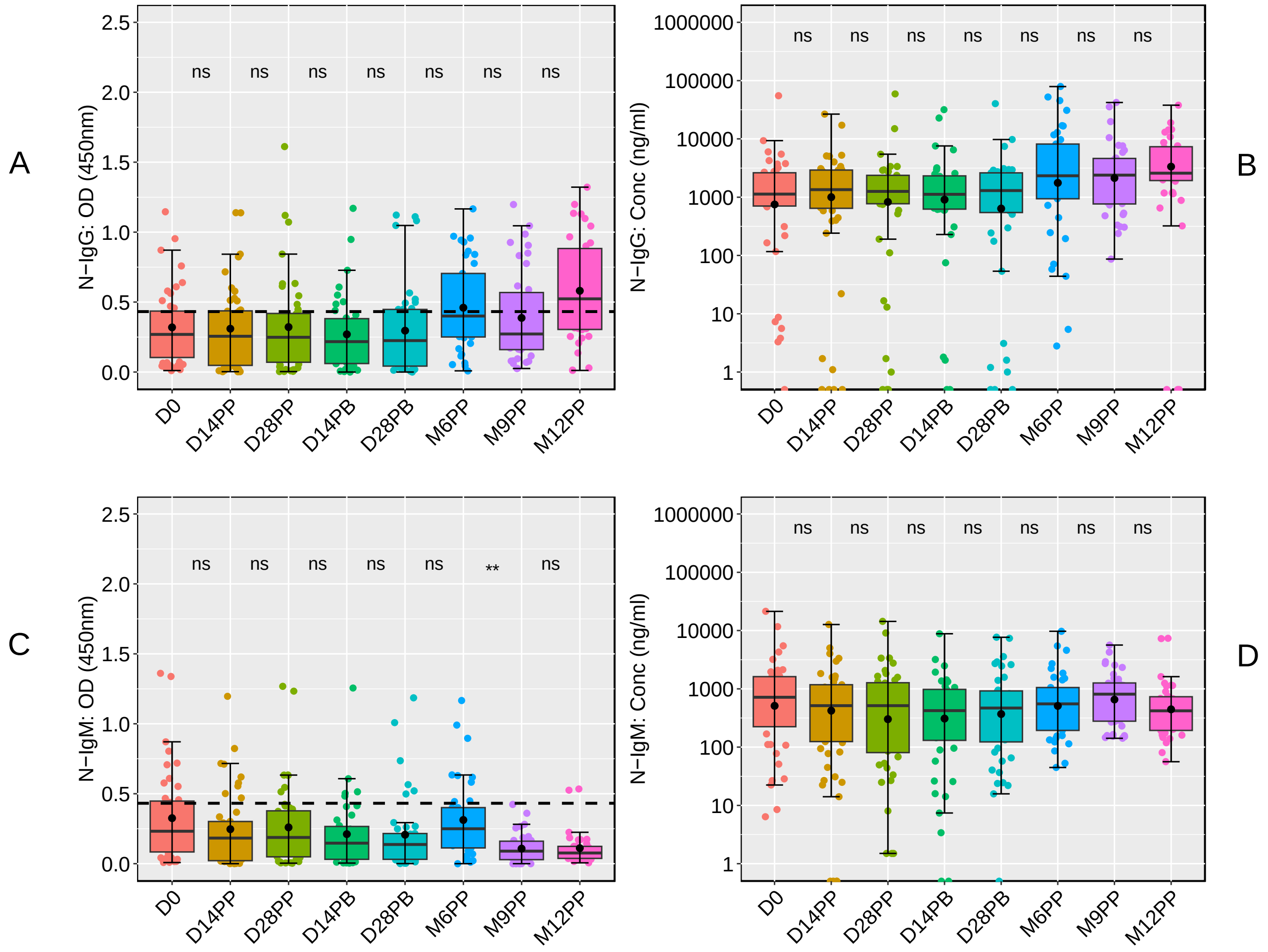

Supplementary Figure 2 illustrates the temporal profile of N-directed antibody responses across 12 months in the cohort. Figures 2A and 2B display Nucleoprotein IgG optical density (OD) and concentrations, while IgM levels are depicted in Figures 2C and 2D. Data distributions are summarised using boxplots, with medians represented by lines, means indicated by black circles, and quartiles marked by box edges. Significance between consecutive time points was determined using an unpaired Wilcoxon test with Bonferroni correction, denoting non-significant differences as "ns" ($p > 0.05$), ** ($p < 0.01$).

Supplementary Figure 3: Box Plots Demonstrating Longitudinal Spike-Directed Antibody Responses Post-Vaccination, Excluding Breakthrough Cases.

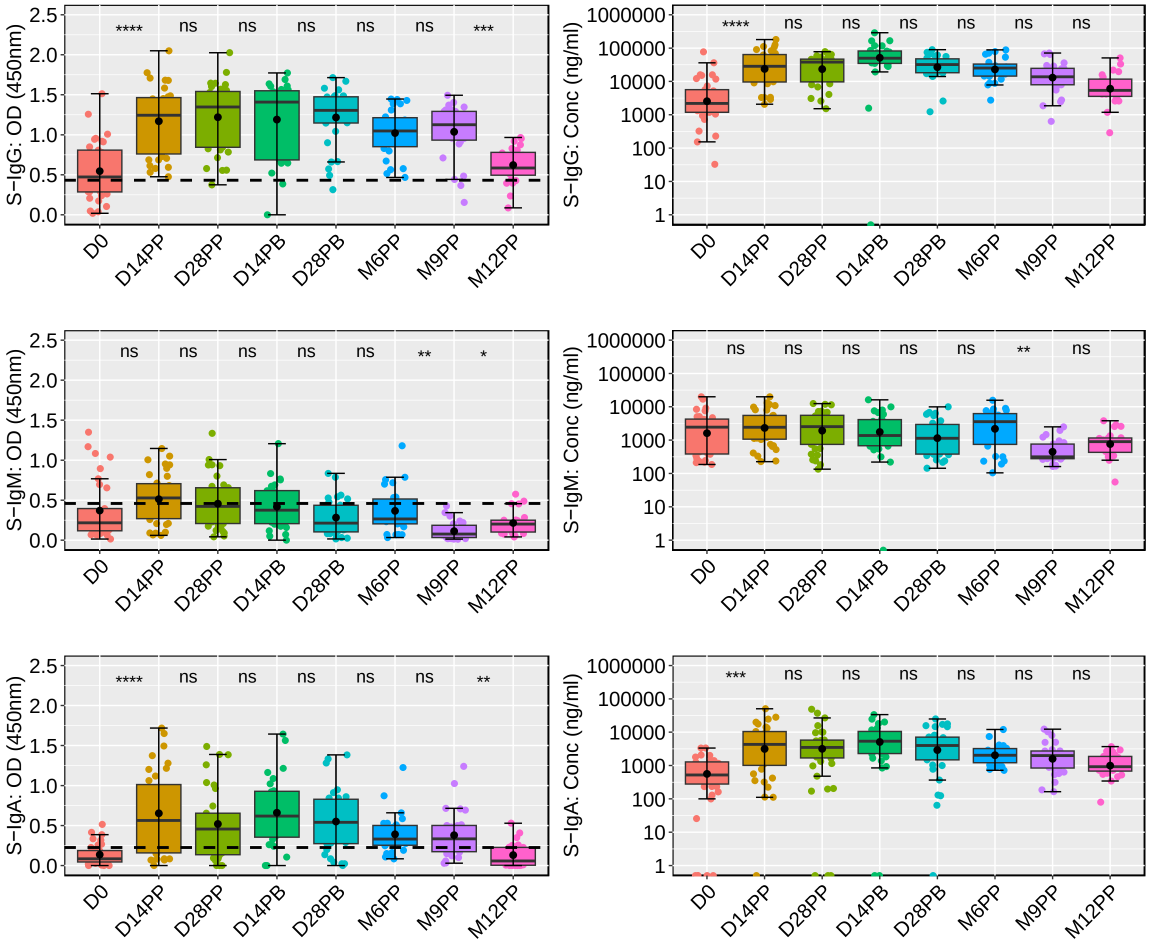

Supplementary Figure 3 depicts the temporal profiles of antibody responses in Pfizer-BioNTech COVID-19 (BNT162b2) vaccine recipients without breakthrough infections over 12 months. Figures 3A and 3B illustrate spike-directed IgG optical density (OD) levels and concentrations, respectively, while Figures 3C and 3D show IgM antibodies, and Figures 3E and 3F represent IgA. Boxplots are used to summarise the data distributions, with medians indicated by lines, means represented by black circles, and quartiles by box edges. Statistical significance between consecutive time points was assessed using an unpaired Wilcoxon test with Bonferroni correction, denoted as follows: (ns: $p > 0.05$; *: $p \leq 0.05$; **: $p < 0.01$; ***: $p < 0.001$; ****: $p < 0.0001$).
